# Hybrid-Aware Model for Senior Wellness Service in Smart Home

**DOI:** 10.3390/s17051182

**Published:** 2017-05-22

**Authors:** Yuchae Jung

**Affiliations:** Sookmyung Women’s University, Seoul 04310, Korea; jungjuri7@sm.ac.kr; Tel.: +82-02-710-9704

**Keywords:** context-aware, inspection service middleware, smart home

## Abstract

Smart home technology with situation-awareness is important for seniors to improve safety and security. With the development of context-aware computing, wearable sensor technology, and ubiquitous computing, it is easier for seniors to manage their health problem in smart home environment. For monitoring senior activity in smart home, wearable, and motion sensors—such as respiration rate (RR), electrocardiography (ECG), body temperature, and blood pressure (BP)—were used for monitoring movements of seniors. For context-awareness, environmental sensors—such as gas, fire, smoke, dust, temperature, and light sensors—were used for senior location data collection. Based on senior activity, senior health status can be classified into positive and negative. Based on senior location and time, senior safety is classified into safe and emergency. In this paper, we propose a hybrid inspection service middleware for monitoring elderly health risk based on senior activity and location. This hybrid-aware model for the detection of abnormal status of seniors has four steps as follows: (1) data collection from biosensors and environmental sensors; (2) monitoring senior location and time of stay in each location using environmental sensors; (3) monitoring senior activity using biometric data; finally, (4) expectation-maximization based decision-making step recommending proper treatment based on a senior health risk ratio.

## 1. Introduction

The concept of smart homes is closely related to home automation and ambient intelligence. The smart home environment (SHE) is enhanced with ‘artificial intelligence’ technology to increase comfort, safety, and quality of life to house holders. The field of health and ambient assisted living is especially focused on technologies for helping elderly people to maintain their autonomy in their daily activities. To achieve a smart environment, senior wellness services must be adapted and context-dependent [1].

The home automation and monitoring technology of biometric data have been developed rapidly with the development of a wireless sensor network, which connects heterogeneous sensors into homes such as biosensors and environmental sensors [2]. These biometric data are highly correlated with environmental situation and it is important to predict changes in vital signs, such as heat rate (HR), pulse rate, respiration rate (RR), systolic blood pressure (SBP), electrocardiogram (ECG), and body temperature. These sensors are wearable or can be installed in elders’ smart home environments. Real-time monitoring using various biosensors can help elders to check their health status by themselves and to update their caregivers with health status in real-time in a smart home environment [3,4]. For personalized healthcare services, all these sensors are linked together in smart home sensor networks and integrate heterogeneous data to predict abnormal health statuses of seniors.

In this paper, we propose a hybrid awareness model for personalized senior healthcare service that can be used for the prediction of abnormal health status using various sensors in a smart home environment. This hybrid-aware model for senior wellness service considers biometric data as well as environmental sensing data. These measurements are used for senior activity and the prediction of senior health status.

This hybrid-aware model has following five modules: (1) monitoring biometric data using biosensors (EEG, ECG, respiration rates, and blood pressure); (2) environmental sensing data collection from environmental sensors (time, temperature, and humidity); (3) zone-aware senior location monitoring; (4) context-aware senior activity monitoring; and (5) decision making for treatment using senior’s health risk ratio based on EM algorithm.

This paper is organized as follows. Section 2 reviews the recent technology for senior wellness services in smart homes. Section 3 introduces an overview of hybrid-aware model for senior wellness service. Section 4 provides the scheme for the hybrid aware model and definition of normal activity patterns, and Section 5 provides result of senior activity based clustering result. Section 6 summarizes our main idea and future direction.

## 2. Related Work

### 2.1. Home Automation Technology

The term ‘smart home’ is one that provides comfort, automation, security, and remote healthcare service to residents with various home automation technologies [5]. For example, AlarmNet is an example of a smart healthcare system, which collects data and analyzes the sensing data to monitor resident’s overall wellness, health status, activities of daily living, and emergency situations. This project considers not only wearable sensor technology, but also considers environmental sensors such as dust, temperature, and light [6]. Many other projects employing smart home technology include various sensor networks, such as biosensors and environmental sensors, that are used for monitoring elders’ health status and assistance of ubiquitous home care service [7,8]. Long-term monitoring of residents’ activity can lead to the prediction of specific disease symptoms—such as stress, cardiovascular issues, cancer, and hypertension [9].

### 2.2. Middleware for Integration of Various Sensor Networks

Smart home technology is based on heterogeneous sensor networks such as wearable and environmental sensors technology for monitoring residents’ health status in real-time. For the integration of various sensor networks, intermediary software layer called middleware is required for interoperability and interface with device drivers [10]. Inspection service middleware provides dynamic support for component management. For the analysis of sensing data, extensive studies have achieved the accuracy of wearable sensors in classifying activities of daily living of elders who are monitored in a smart home environment. Machine learning algorithms—such as support vector machines, Bayesian classification, and neural networks—are widely used to detect normal activity, as well as abnormal activity.

### 2.3. Ubiquitous Home Care Service for the Assistance of Senior

The area of home automation technology is applied to ubiquitous assistance for elders and disabilities in daily life. For example, smart home technology is also important for the detection of abnormal situations such as fire, medical emergency, and accidents for safety purposes [9,10]. For the detection of abnormal situations, smart homes equipped with surveillance camera, healthcare monitoring systems, motion sensors, and alarm systems. The aim of smart home technology is to provide safety, security, home care, and energy efficiency to the residents by detection of abnormal activity with contextual awareness technologies. However, current smart homecare service lacks the interoperability and integration properties between heterogeneous sensor data [11]. Our proposed hybrid-aware model can improve interoperability by providing inspection service middleware for the integration of senior biometric data with environmental information for smart homecare service.

## 3. The Overview of Hybrid-Aware Model for Senior Wellness Service

**Figure 1:**
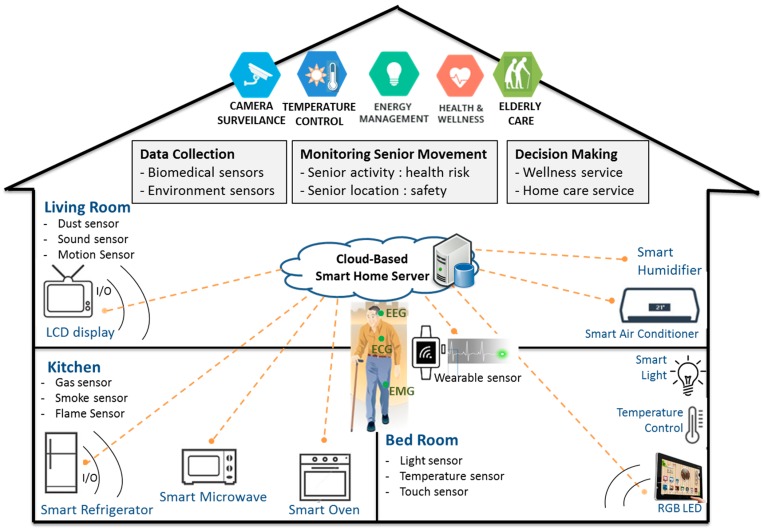
The overview of hybrid awareness model for senior wellness service in smart home. The hybrid awareness model consists of data collection from bio-sensors and environmental sensors, monitoring senior activity and location, and decision-making. A cloud-based smart home server collects sensing data and uses it for analysis with various machine learning algorithms for smart home care service.

The overview of the hybrid-aware model for senior wellness service in smart homes is shown in Figure 1. In smart home systems, there are five modules (sensing biometric data, sensing environmental information, location monitoring, activity monitoring, and decision-making) for senior wellness service.

In the first step, elders’ biometric data are monitored by multimodal biosensors (EEG, ECG, RR, and BP) and situational information is checked with environmental sensors (gas, dust, CO, temperature, sound, motion, and touch). In the zone-aware location monitoring step, senior’s location (living room, kitchen, toilet, and bed room) is checked with smart electronics for monitoring senior location. In the context-aware activity monitoring step, elders’ movement in smart home is checked for senior health status. We simply defined senior movements as housework, rest, exercise, and sleeping based on the location and time of stay in each location for monitoring senior health status. We also generated rules for the classification of normal health status for elders based on senior activity and location in smart home. Finally, this sensing information is stored in the smart home server and analyzed with various machine learning algorithms for clinical decision support and senior home care service.

## 4. The Scheme of Proposed Hybrid-Aware Model

### 4.1. Hybrid-Aware Model for Senior Home-Care Service in User’s Context

**Figure 2:**
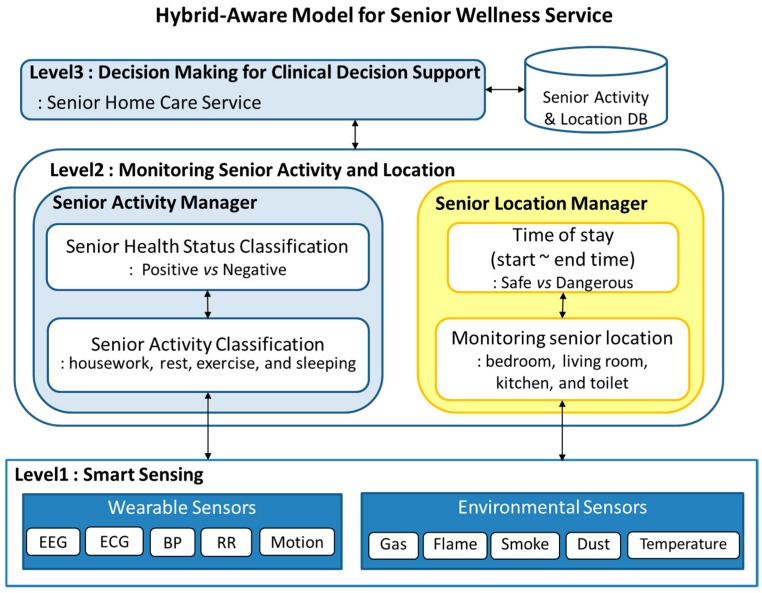
The flow of hybrid-aware model for senior wellness service (1) Level 1: Data collection from wearable sensors and environmental sensors, and smart device. (2) Level 2: monitoring senior activity and location. (3) Level 3: decision-making for clinical decision support for senior home care service.

Figure 2 represents the work-flow of hybrid-aware model for monitoring senior health status in smart home environment. This hybrid-aware model has five major steps as follows: (1) data collection from biomedical wearable sensors; (2) collection of situational information using environmental sensors; (3) senior location manager monitors senior location from environmental sensors and classifies the location as bedroom, living room, kitchen, dining room, or toilet; (4) from wearable sensors, the activity manager detects senior activity and classifies it as housework, rest, exercise, and sleeping; (5) EM-based decision-making step recommends the proper home care service based on senior health status as well as clinical decision support for emergency health situations.

The first step is the collection of sensing data from wearable sensors, smart electronics, and environmental sensors in smart home. In this step, sensing data from various sensors is collected and stored in smart home servers for the generation rule DB. By monitoring senior activity and location, biometric data, inference engine classify senior health risk ratio into two types (normal and abnormal). Depending on the senior health risk ratio, proper activities are recommended using the EM algorithm. The decision-making step recommends proper activities such as music therapy, exercise, and hospital checkup.

### 4.2. Database Scheme for Monitoring Senior Health Status

**Figure 3:**
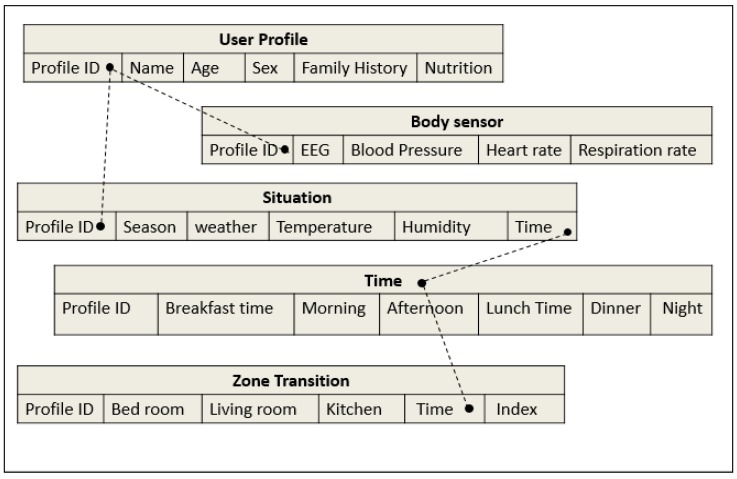
The database scheme for the storage of bio-sensing data of senior health status monitoring.

We designed a flexible database scheme that incorporates user profile with sensing data and situational information as shown in Figure 3. These database schema are designed for monitoring senior activity and time of stay and zone transition in a smart home. A user profile table consists of a key, a profile ID, which again may serve as a pointer to the bio-sensing data and situation tables.

### 4.3. The Collection of Biometric Data Using Multimodal Biosensors

Multi-modal biosensors are used for the data collection. Four health parameters (EEG, BP, HR, and RR), which is known to change with aging are selected as Table 1. In order to simplify biometric data, fuzzy logic is used to categorize sensing data as linguistic variables. For the detection of sensing data patterns, the monitoring step defines the criteria of bio-sensing data into three levels (low, normal, and high) in Table 1. Blood pressure and respiration rates are known to increase with aging and heart rate is known to decrease with aging [12]. For detection of abnormal senior health status, normal biometric data is defined as Table 1 for the prediction of senior health risk ratio.

### 4.4. Monitoring Senior Biometric Sensing and Activity Pattern Depending on Time

To obtain the senior location data, location manager is monitoring senior movement using smart electronics and environmental sensors. In each location, activity log was monitored for the detection of time of stay. The relationship between biometric data changes and activity pattern is defined as the categories in Table 2 based on the daily activity log and previous studies [13,14].

### 4.5. The Classification of Normal and Abnormal Activity

For monitoring senior activity in each location, we generated rules for the detection of normal and abnormal health status of elders (as shown in Table 3). Based on routine activity in knowledge database, abnormal status (more toileting, more sleeping, and no biometric data checking) is defined as Table 2 based on daily log for checking a senior’s medical emergency situation.

### 4.6. The Definition of Senior Health Risk Based on Activity, Location, and Biometric Data 

We have randomly generated 100 samples for 10-min single-user scenarios in a smart home environment in Table 4. Based on senior location, activity and biometric data, senior health risk is defined in this step. Four features (EEG, BP, HR, and RR) known to be associated with aging are selected [12,13] and senior health risk ratio is classified into four steps (normal, low, middle, and high). The accumulated senior health risk ratio is used for clinical decision-making.

### 4.7. EM Algorithm Based Decision-Making for Senior Home Care Services

Based on our previous studies [15,16], we propose a hybrid-aware model based on senior health risk ratio, proper treatment was recommended based on an Expectation Maximization (EM) algorithm. EM is an algorithm that can calculate maximum likelihood estimates from incomplete data [17]. An EM algorithm is composed of a two-step process: the expectation (E-step) and the maximization step (M-step). The EM algorithm alternates between E-step and M-step until the maximized risk ratio is bigger than ε. Latent variables (maximized senior health risk ratios) are variables that are not directly observed but are rather inferred from other variables such as context-aware senior health status and zone-aware environmental information. The local optimality can be guaranteed by the convergence of the EM algorithm. In the M-step, by using the SHR, proper wellness contents (music therapy, exercise, and hospital checkup) are recommended for treatment and clinical decision-making.

As described Algorithm 1, the senior health risk EM algorithm composed of two steps, and alternates between E-step and M-step. In E-step, the input variables are repeatedly updated to understand elders’ health status in the smart home. The maximized senior health risk is repeatedly calculated with a cumulative senior health risk ratio. In M-step, by using the SHR, a proper wellness contents (music therapy, exercise, and hospital checkup) are recommended for treatment and clinical decision-making.

**Algorithm 1.** Senior health risk ratio EM for decision making based on health status**Input:**  Monitoring Senior Activity (watching TV, rest, exercise, sleeping, and lunch)  Monitoring Senior Location (living room, kitchen, bedroom and toilet)  Monitoring Senior Biometrics (EEG, BP, HR, and HRV)  Context-aware SeniorHealthStatus (Normal, Low, Middle and High)  current LogLikelihood  ←  ∞ **repeat**  prevSHR  ←  current SeniorHealthRisk     calculate SeniorHealthRiskRatio **until**   check max SeniorHealthRiskRatio < ε     If (EEG is *Alpha*, BP is *normal*, and HRV is *normal*) then (SHR is *Low*)   elseIf (EEG is *Beta*, BP is *increased* and HRV *is normal*) then (SHR is *Middle*)     else (EEG is *Beta*, BP is *increased* and HRV is *decreased*) then (SHR is *High*)**Output:**   Recommend wellness contents based on SeniorHealthRisk   If (SHR is *Normal*) then (recommend regular work)   If (SHR is *Low*) then (recommend *Music therapy*)   If (SHR is *Middle*) then (recommend *Exercise*)   If (SHR is *High*) then (recommend *Hospital Checkup*)

## 5. Experimental Results

### 5.1. The Analysis of Senior Biometric Data with Situation Awareness

**Figure 4:**
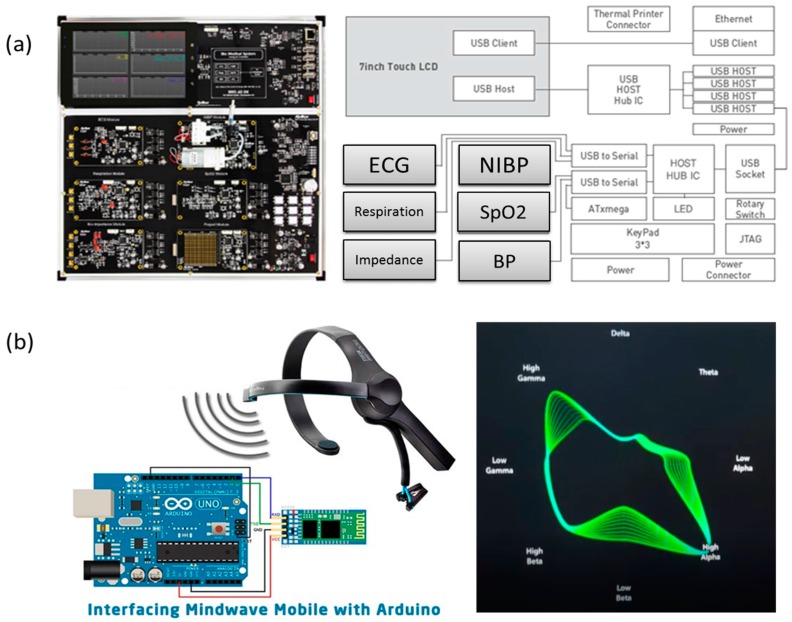
Wearable sensors for biometric data collection. (**a**) The multimodal biomedical sensors for biometrics sensing and (**b**) EEG sensors for brainwave detection.

Using BMS-AE-DK (biomedical system development kit) biosensors in Figure 4a (http://www.hybus.net/goods/view.asp?idx=89&category=25) from HyBus, biometric data—such as electrocardiogram (ECG), respiration rate (RR), SpO_2_, systolic blood pressure (SBP), and diastolic blood pressure (DBP)—are repeatedly measured for data collection. For brainwave detection, Neurosky mindwave mobile was used as Figure 4b.

### 5.2. The Analysis of Activity Pattern and Biometric Data

**Figure 5:**
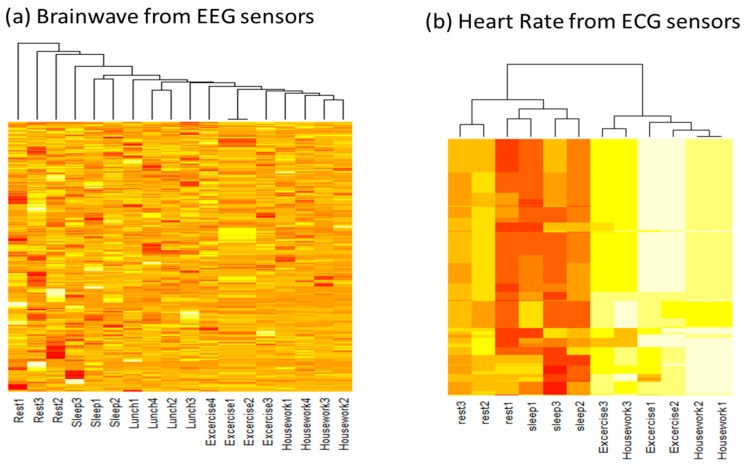
Biometric data changes during different senior activities. (**a**) The brainwave data from EEG sensors during five different activities and (**b**) heart rate from ECG sensors during four activities.

For the detection of biometric data changes depending on senior activity patterns, brainwaves were measured repeatedly during rest, sleeping, lunch, exercise, and housework as Figure 5a. Unsupervised clustering method is used for the detection of relationship between brainwave changes during different senior activity. During rest and sleeping activities, alpha wave was dominant, while exercise and housework showed beta waves. Senior heart rate was also repeatedly measured from ECG sensors as Figure 5b. During rest and sleeping activities, heart rate was lower (60–90 beats per min) compared to exercise and housework activities (95–125 beats per min). Our hybrid-aware model can distinguish senior activity patterns by measuring brainwave and biometric data (heart rate and blood pressure).

### 5.3. The Correlation between DBP, BMI, and FBS

**Figure 6:**
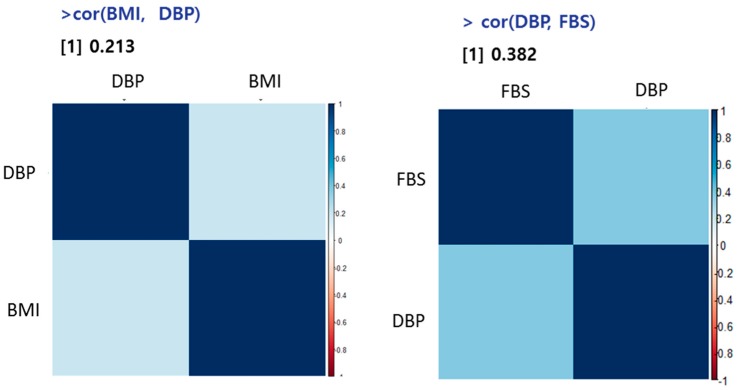
The Pearson’s correlation analysis between BMI and DBP vs. DBP and FBS.

Age, fasting blood glucose (FBS), and body mass index (BMI) information are also collected from 10 male and 12 females. The Pearson’s correlation analysis between BMI, DBP, and fasting blood sugar (FBS) was performed with R package. As shown in Figure 6, BMI and blood pressure showed high correlation (*R* = 0.21) and diastolic blood pressure and FBS also showed high correlation (*R* = 0.38). It is known that blood pressure typically increases with age, especially once one passing middle age [12]. According to the National Heart, Lung, and Blood Institute, someone with healthy blood pressure at age 60 has a 90% chance of developing hypertension later in life. Aging leads to a general weakening of the heart, which can cause a slow heart rate.

## 6. Conclusions

This paper outlines a hybrid-aware model for the prediction of senior health status by monitoring senior activity and location in the smart home. Senior activity is monitored in each location considering the time of stay for monitoring senior health status. Based on senior health status, SHR is classified into four levels (normal, low, middle, and high) and proper treatment is recommended using an EM algorithm. This hybrid-aware model can support healthcare professionals in clinical decision-making through CDSS (Clinical Decision Support System). This hybrid-aware model also can help to detect abnormal health status to prevent medical emergencies using situation-awareness technology.

## Figures and Tables

**Table 1:** The definition of normal range of biometric data.

Sensor Type	Low	Normal	High	Senior
Electroencephalogram (EEG) (Hz)	Delta (1–3)	Alpha (8–13)	Beta (14–30)	
Blood pressure (BP) (bpm)	<90	90–120	>120	increase with aging
Heart rate (HR) (bpm)	20–60	50–100	90–120	decrease with aging
Respiration rate (RR) (bpm)	5–10	7–20	15–25	increase with aging
O2 saturation	80–92	93–99	100%	
Blood sugar		4.0–5.9 mmol/L (before meal)	>7.8 mmol/L (after meal)	
Temperature		36.1 °C	>37.9 °C	
Respiration rate	<9	9–20 bpm	>21 bpm	

**Table 2:** The relationship between biometric data and activity pattern in each location.

Activity	EEG	Biometrics	Location	Time (s)
Sleeping	Delta wave (increase)	BP, RR (decrease)	Bedroom	20,374
Rest	Alpha wave (increase)	HR (decrease)	Living room	836
Exercise	Beta wave (increase)	HR, RR (increase)	Living room	1244
Lunch	Theta (decrease)	Blood Sugar (increase)	Dining room	1146
Housework	Bta wave (increase)	BP, HR (increase)	Kitchen	2455
Toileting		BP (increase)	Restroom	1035
…	…	…	…	…

**Table 3:** The classification of senior normal activity and abnormal activity.

Type	Normal	Abnormal	Observation
Activity	Sleeping–cooking–rest–exercise–lunch	Sleeping–toileting–breakfast–toileting–housework–lunch	No exercise, more toileting
Activity	Sleeping–toileting–check biosensor–breakfast–rest	Sleeping–toileting–breakfast–rest	Biosensors are not checked before breakfast
Activity	Lunch–housework–dinner–rest–sleeping	Lunch–housework–sleeping–rest–housework–dinner	Sleeping before dinner
Location	Bedroom–toileting–living room–kitchen–living room–toilet–bedroom	Bedroom–toilet–living room–toilet–kitchen–toilet–bedroom	More frequent toilet visits

**Table 4:** The classification of senior health risk based on activity, location, and biometric data.

Activity	Heart Rate	Blood Pressure	EEG	Location	Senior Health Risk
Watching TV	High	High	Beta	Living Room	High
Rest	High	Normal	Beta	Living Room	Middle
Exercise	High	Normal	Alpha	Kitchen	Low
Lunch	Normal	Normal	Alpha	Living Room	Normal
Sleeping	Normal	Normal	Theta	Bedroom	Normal

## References

[1] Cook D.J. (2009). Multi-agent smart environments. J. Ambient Intell. Smart Environ..

[2] Marco Z.Z., Bhaskar K. (2003). Integrating Future Large-Scale Wireless Sensor Networks with the Internet.

[3] Juan A., Vic C., Diane C., Achilles K., Ichiro S. (2013). Intelligent Environments: A manifesto. Human-Centric Computing and Information Sciences.

[4] Holger N., Michael C. The Semantic Sensor Network Ontology: A Generic Language to Describe Sensor Assets. Proceedings of the 12th AGILE International Conference on Geographic Information Science.

[5] Saisakul C., Anthony S.A., Hongnian Y. Perception of Smart Home Technologies to Assist Elderly People. Proceedings of the 4th International Conference on Software, Knowledge Information Management and Applications (SKIMA2010).

[6] Wood A., Virone G., Doan T., Cao Q., Selavo L., Wu Y., Fang L., He Z., Lin S., Stankovic J. (2006). ALARM-NET: Wireless Sensor Networks for Assisted-Living and Residential Monitoring.

[7] Hadi B., Mobyen U.A., Amy L. (2013). Data Mining for Wearable Sensors in Health Monitoring Systems: A Review of Recent Trends and Challenges. Sensors.

[8] Hemant G., Subhas M., Xiang G., Nagender S. (2015). WSN- and IOT-Based Smart Homes and Their Extension to Smart Building. Sensors.

[9] Zanifa O., Fredrick M., Bing W., Ciaran D. (2011). Ubiquitous Healthcare Information System: Assessment of its Impacts to Patient’s Information. Int. J. Innov. Sci. Res..

[10] Badica C., Brezovan M., Badica A. An Overview of Smart Home Environments: Architectures, Technologies and Applications. Proceedings of the Balkan Conference in Informatics (BCI’13).

[11] Mukhtiar M., Stefan R.W., Christian F.P., Femina H.A.B., Finn O.H. (2014). Ambient Assisted Living Healthcare Frameworks, Platforms, Standards, and Quality Attributes. Sensors.

[12] Chester J.G. (2011). Vital signs in older patients: age-related changes. J. Am. Med. Dir. Assoc..

[13] Wang X., Nie D., Lu B. EEG-based emotion recognition using frequency domain features and support vector machines. Proceedings of the 2011 International Conference on Neural Information Processing (ICONIP 2011).

[14] Campbell G. (2010). EEG Recording and Analysis for Sleep Research. Curr. Protoc. Neurosci..

[15] Jung Y., Yoon Y. (2016). Multi-level assessment model for wellness service based on human mental stress level. Multimedia Tools and Applications. Multimed. Tools Appl..

[16] Jung Y., Yoon Y. (2015). Ontology model for wellness contents recommendation based on risk ratio EM. Proced. Comput. Sci..

[17] Logothetisa A., Krishnamurthyb V., Holst J. (2002). A Bayesian EM algorithm for optimal tracking of a maneuvering target in clutter. Signal Process..

